# Obstacle Recognition Based on Machine Learning for On-Chip LiDAR Sensors in a Cyber-Physical System

**DOI:** 10.3390/s17092109

**Published:** 2017-09-14

**Authors:** Fernando Castaño, Gerardo Beruvides, Rodolfo E. Haber, Antonio Artuñedo

**Affiliations:** Centre for Automation and Robotics, Technical University of Madrid-Spanish National Research Council (UPM-CSIC), Ctra. Campo Real Km. 0.2, Arganda del Rey 28500, Spain; gerardo.beruvides@car.upm-csic.es (G.B.); rodolfo.haber@car.upm-csic.es (R.E.H.); antonio.artunedo@car.upm-csic.es (A.A.)

**Keywords:** sensor-in-the-loop, co-simulation framework, virtual cyber-physical system, on-chip LiDAR, obstacle recognition library

## Abstract

Collision avoidance is an important feature in advanced driver-assistance systems, aimed at providing correct, timely and reliable warnings before an imminent collision (with objects, vehicles, pedestrians, etc.). The obstacle recognition library is designed and implemented to address the design and evaluation of obstacle detection in a transportation cyber-physical system. The library is integrated into a co-simulation framework that is supported on the interaction between SCANeR software and Matlab/Simulink. From the best of the authors’ knowledge, two main contributions are reported in this paper. Firstly, the modelling and simulation of virtual on-chip light detection and ranging sensors in a cyber-physical system, for traffic scenarios, is presented. The cyber-physical system is designed and implemented in SCANeR. Secondly, three specific artificial intelligence-based methods for obstacle recognition libraries are also designed and applied using a sensory information database provided by SCANeR. The computational library has three methods for obstacle detection: a multi-layer perceptron neural network, a self-organization map and a support vector machine. Finally, a comparison among these methods under different weather conditions is presented, with very promising results in terms of accuracy. The best results are achieved using the multi-layer perceptron in sunny and foggy conditions, the support vector machine in rainy conditions and the self-organized map in snowy conditions.

## 1. Introduction

Recent developments demonstrate an increasing efficiency, availability and affordability of sensors, data acquisition systems and computer networks [1]. Cyber-physical systems (CPSs) are still growing in different engineering fields, supporting applications across industries such as manufacturing, healthcare, electric power grids, agriculture and transportation. Nowadays, dozens of contributions are reported in the literature addressing key CPS issues [2], from connecting topologies to cognitive and self-configuration layers. The computational requirements in relation to operating systems, programming languages, user interfaces and networking technologies have become more sophisticated in relation to software managing, information flow control, error control, redundancy, reliability and latency in heterogeneous global networks [3]. Furthermore, the knowledge acquisition, learning [4] and its transformation into physical actions to help machines in decision-making activities are priorities in the paradigm of CPSs. 

One of the main applications for sensing systems and CPSs is in advanced driver-assistance systems (ADAS) and autonomous vehicles (AV). According to the report of the company Accenture LLP, for the next ten years and beyond, the key areas into the automotive vehicle industry are: (i) cyber security; (ii) product liability for sensors and software and/or algorithms; and (iii) insuring AV infrastructure [5]. In particular, the sensor-in-the-loop system has been reported in several studies, with promising results in relation to high accuracy and precise six-degrees-of-freedom position information for real-time navigation [6]. Many vision-based navigation algorithms are also available for AV systems. Among them, the Light Detection and Ranging (LiDAR) system and stereo-vision cameras are widely used in computer vision for autonomous vehicle applications [7]. 

**Figure 1:**
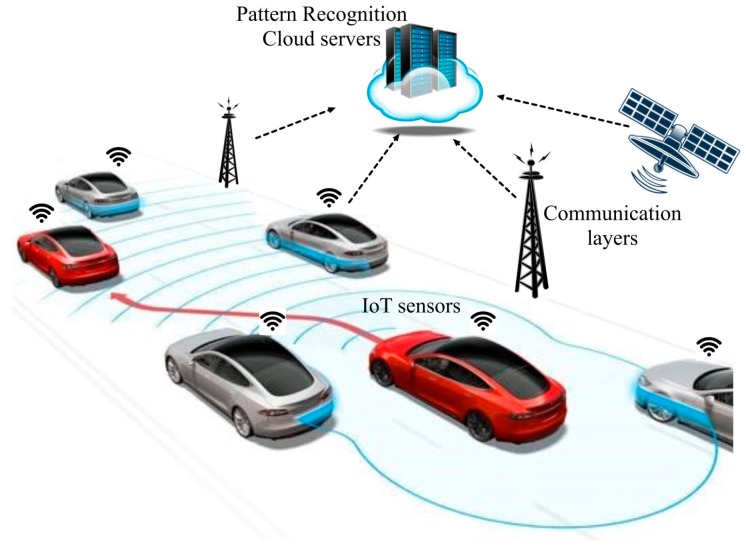
Conceptual vehicle-road-environment interactions.

Obstacle recognition multi-layer architectures to represent the common patterns of roads, lane markings, traffic signals, vehicles, pedestrians and so on are a priority line for researchers and car fabricants (Figure 1). Nowadays, many classifiers rely on machine-learning approaches to exploit data redundancy and abundance to find out patterns, trends and relations amongst input attributes and class labels [8,9,10]. Within obstacle-recognition techniques, vector support machines have been widely applied for classification and regression problems [11]. An interesting application using machine learning for pedestrian detection in autonomous vehicles based on High Definition (HD) 3DLiDAR is reported in [12], providing more accurate data to be successfully used in any kind of lighting conditions.

On the other hand, co-simulation frameworks take into account physical dynamics, control software, computational platforms and communication networks, which is crucial for designing CPSs for autonomous driving [13]. Co-simulation is essential for CPSs due to virtual prototyping, and is capable of properly emulating actor–sensor nodes with their own hardware specifications [14]. Moreover, virtual prototyping can take advantage of different modelling languages and tools, and integrate them together for evaluating the behaviour of CPSs. For example, processing elements with real-time operating systems [15], communication systems, sensors, actuators, model transformations to the final virtual prototype [16], and localization error estimation and compensation [17] can be efficiently represented and modelled.

This paper presents the design and application of an obstacle-recognition library with three artificial intelligence-based methods for collision avoidance in driving-assistance scenarios. This library is integrated into a co-simulation framework for modelling and simulating a virtual sensor network in a cyber-physical system. From the best of the authors’ knowledge, three contributions are reported in this paper. Firstly, a simulation framework of virtual sensors for improving the accuracy of on-chip LiDAR sensors is presented. This simulation framework enables in-parallel data from connected sensor networks in a CPS to be obtained. Secondly, three artificial intelligence-based methods—a multilayer perceptron, a self-organized map and a support vector machine—are included in the library. These methods were selected due to their solid mathematical foundations, demonstrated suitability for modelling in complex scenarios and worldwide successful applications reported in the literature. Finally, a comparative study among these techniques for detecting obstacles is presented, in order to assess their performance in different weather conditions. 

In addition, an obstacle database in the CPS for driving assistance was generated from the SCANeR simulator to assess the on-chip LiDAR sensor accuracy under different weather conditions. The on-chip LiDAR concept has led to a great technological challenge with regard to sensor networks in CPSs. Due to its limited measurement range and field of view, it is necessary to obtain in-parallel data from other connected sensors to set a more accurate scan of the whole environment. 

This paper addresses topics of high relevance to Europe. One of the motivations of this work is the need to validate work previously developed in our institution in the frame of the EMC2 European project [18]. The ENABLE S3 project also corroborates the priorities of these research topics in Europe [19]. Moreover, the proposed co-simulation system will be embedded and validated in real driving environments as part of the contributions to the IoSENSE project [20]. 

The paper consists of five sections. Following this introduction, the second section explains the co-simulation framework composed by two modules: SCANeR and Simulink. Subsequently, a case study based on the interaction between the SCANeR simulator and MatLab in a driving assistance scenario is explained in Section 3, as well as the first preliminary results obtained. After that, experimental results and a discussion by means of a comparative study are addressed in Section 4. Finally, the conclusions and future research steps are presented.

## 2. CPS Co-Simulation Framework Description

The CPS co-simulation framework mainly consists of a computer-aided system to enable an efficient interaction between SCANeR studio and Matlab/Simulink. A set of computational procedures is in charge of adapting and transferring sensory information from SCANeR to MatLab and vice versa. The transfer of data is carried out by means of different functionalities available in SCANeR studio. 

The co-simulation framework is implemented using the software developer kit of SCANeR. For example, some of the available functions are created with C/C++, C#, Labview, Matlab/Simulink or Python. LiDAR, 3D stereo vison cameras and GPS sensors can be emulated with this software. 

**Figure 2:**
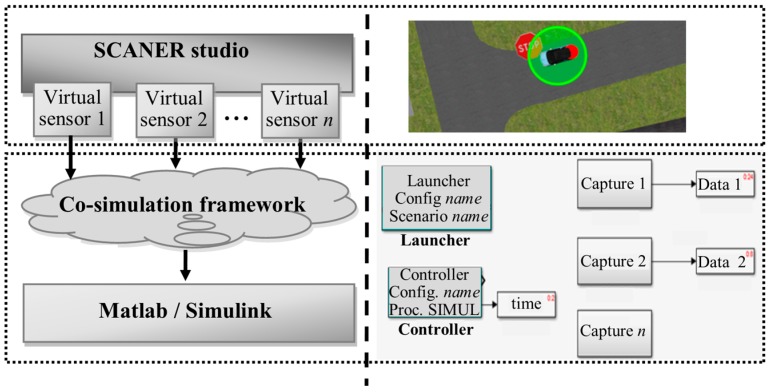
Co-simulation framework architecture.

A two-module co-simulation system is introduced in this work. The first module is used to generate multiple 3D traffic scenarios composed by different nodes that belong to a sensor network, using SCANeR. The second module with three specific artificial intelligence-based methods (i.e., artificial neural network, self-organized map and support vector machine) is implemented in Matlab/Simulink. A classifier is then derived from data cloud points given by virtual sensors in the CPS (Figure 2). Matlab/Simulink can easily interact with SCANeR studio, even in real time, thought a software development kit (SDK) module. The main goal of the classifier system is the detection of multiple obstacles in traffic scenarios. 

### 2.1. SCANeR Studio Module

SCANeR is a simulation engine for automotive applications in a virtual environment. The functionalities of SCANeR can be extended by an interface with third party modules by means of the SDK tool.

#### 2.1.1. 3D Traffic Scenario

**Figure 3:**
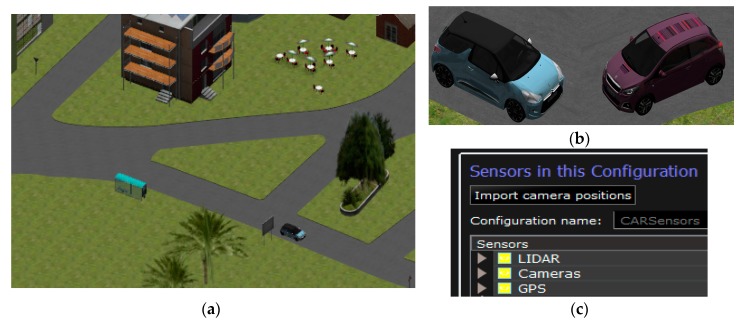
Traffic scenario in SCANeR. (**a**) Aerial view of simulation scenario; (**b**) vehicle models and virtual CPS; (**c**) sensor configuration.

A 3D traffic scenario can be created in order to simulate the behaviour of virtual sensor networks in CPSs for driver-assistance systems. For example, a simple scenario can be composed of elements that represent an urban environment (Figure 3a).

Moreover, each vehicle model represented in this simulation tool can be equipped with specific sensors and actuators models (Figure 3b). Furthermore, a control architecture can also be included in this simulation tool. For example, this architecture can be based on a fuzzy logic controller that manages individual actions on the throttle, brake and steering wheel, from sensory information [21].

#### 2.1.2. Virtual Models of CPS Components

Different sensors can be represented in order to equip vehicles with these on-board sensors (Figure 3c). Therefore, sensor networks can be created in order to emulate the behaviour of virtual sensors or actuators in the CPS. The co-simulation enables new designs, including new features. For example, key steps such as pre-processing (invalid scan points), segmentation (clustered point clouds), and feature extraction (identify features for the individual scans) and classification (object-type detection) for the LiDAR sensor can be designed, conditioned and tailored. 

### 2.2. Matlab/Simulink Module

The second module consists of a library and classification models, implemented in Matlab/Simulink, in order to identify different object types. The framework is composed by a knowledge database and a library with three artificial intelligence-based methods by default (i.e., artificial neural network, self-organized map and support vector machine), although this library can be enriched at runtime from data received by all nodes that make up the virtual sensor network. The functional blocks are distributed in different nodes according to their functions. The distributed mobile nodes are in charge of capturing sensory data and run the classification with the required accuracy, whereas the main static node incorporates the generated runtime model, the library and the knowledge database.

**Figure 4:**
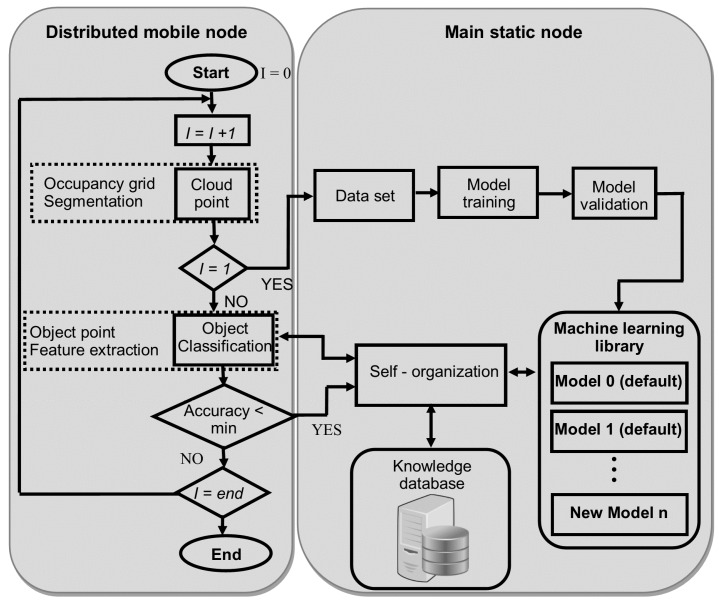
Block diagram of classification methodology procedure for object-type identification.

The flow diagram of the procedure is shown in Figure 4. The distributed mobile node is also composed by the “cloud point” block that includes the occupancy grid generation and segmentation of ground plane and nearby obstacles. These nodes also include the classification block for object point detection and feature extraction. 

On the other hand, the main static node contains the default library with some classification models. Later on, the library can be enriched from the process simulation. New traffic situations are generated providing new clouds of points (environment information) in each interaction between sensors and the obstacle-detection procedure. Based on this continuous information flow and the previous classification (knowledge database), the library executes a parallel learning procedure for all the classification models to obtain a personalized setting for each particular scenario. Finally, once a new best configuration is yielded, the corresponding classifier in the distributed mode is then updated.

The decision to select the best classifier model included in the library is carried out by all virtual nodes, although some of them have not yet individually detected a decrease in their particular classification performance metrics. The performance metric selected to evaluate the accuracy in obstacle detection is the correct classify samples or correct rate (CCR). This performance index is computed from the best estimation about the classification of each instance in the test set. Then, the predicted classifications are compared to the real classification values to assess the actual accuracy. This also generates a knowledge database with the performance of classifier models. In this way, the best models for each weather condition can be recorded. Three techniques implemented in the library are the multi-layer perceptron neural network (MLP) [22], self-organized map (SOM) [23] and support vector machine (SVM) [24]. 

MLP is one of the pioneering and most studied topology of artificial neural networks successfully applied in pattern recognition and modelling. The most popular supervised learning algorithm is the error backpropagation. The operation in a perceptron is described by the formula:

(1) $$y=f\left(\sum_{i=1}^{n}Wp_{i}x_{i}+bp\right),$$

where *f* is a discontinuous step function; *n* is number of inputs in a neuron; *x* the input signals; *Wp* the synaptic weights; *bp* the threshold value or *bias*; and *y* the neuron output value.

For the training algorithm, the steepest descent is applied:

(2) $$\Delta Wp^{n}=-\alpha \frac{\partial L}{\partial Wp^{n}},$$

where ∆*Wp^n^* is the *n*-th weight update and α is the learning rate. This process is repeated until some stopping criteria are met. A major problem with gradient descent is that it easily gets stuck in local minima. This can be mitigated by the addition of a momentum term, which effectively adds inertia to the motion of the algorithm through weight space, thereby speeding up convergence and avoiding local minima [25]:

(3) $$\Delta Wp^{n}=m\Delta Wp^{n-1}-\alpha \frac{\partial L}{\partial Wp^{n}},$$

where *m* is the momentum parameter.

The self-organizing map belongs to unsupervised learning methods, that is, there are no explicit target outputs associated with each input, and the goal is to build representations of the input that can be used for decision making. The mapping of the SOM is done by feature vectors associated with each unit, $Wsom_{i}=(Wsom_{i}^{1},Wsom_{i}^{2},\ldots ,Wsom_{i}^{n})$. A sequential description of how to train a Kohonen SOM is as follows [26]:

1. Initialize all weights randomly;
2. Choose operating point (OP) randomly in the training set;
3. Select the winning output unit as the one with the largest similarity measure between all weight vectors and the operating point *x*. The winning unit satisfies the following equation:
  (4) $$\left|x-Wsom_{c}\right|=\min\left|x-Wsom_{i}\right|.$$
4. Define the neighbourhood of the winner, by using a neighbourhood function $\Omega_{c}(i)$ around a winning unit *c*. For instance, the Gaussian function can be used as the neighbourhood function as follows:
  (5) $$\Omega_{c}(i)=\exp^{\left(-\frac{\left|p_{i}-p_{c}\right|}{2\sigma^{2}}\right)},$$
  where *p_i_* and *p_c_* are the positions of the output units *i* and *c*, respectively, and *σ* reflects the scope of the neighbourhood. After the definition of the neighbourhood function, the weight vector *ω_c_* of the selected neuron and the weight vectors *ω_i_* of its neighbours are updated according to the following formula:
  (6) $$\Delta Wsomi=\alpha \cdot \Omega_{c}(i)\cdot (x-Wsom_{i}).$$
5. Finally, if the neighbourhood function is bigger than the allowed error, go to 2; else, stop.

To achieve convergence, the learning rate and the width of the neighbourhood of the winner neuron must shrink to zero with time. A main problem of the SOM algorithm is the fact that the number of training steps of the convergence phase has to be fixed a priori, and therefore, must be set to a large value in order to ensure convergence.

Finally, support vector machines are a group of supervised learning methods strongly cited in signal and image classification tasks. A kernel function (*K*) $K:{\mathbb{R}}^{m}\times {\mathbb{R}}^{m}\to \mathbb{R}$ can be described as:

(7) $$K(X_{i},X_{j})=\phi {\left(X_{i}\right)}^{t}\phi (X_{j}),$$

where *X* is a pattern text and *φ* the mapping representation.

Given a matrix *X* the classification function can be represented:

(8) $$K(X_{i},X_{j})=\sum_{X_{i}\in S}Ws^{t}\phi (X_{j})+bs,$$

where *S* is the set of supported vectors, *Ws* is the weight and *bs* the support bias.

In particular, the Gaussian kernel is one of the most reported SVM techniques in the literature for nonlinear decision-boundary spaces.

(9) $$K(X_{i},X_{j})=e^{-\frac{{\left\|xi-xj\right\|}^{2}}{\sigma^{2}}}.$$

Without doubt, these methods are powerful tools to carry out pattern classification for obstacle detection [27]. Nevertheless, the design, simulation and implementation of effective solutions beyond the academia are still challenging for researchers and engineers. Indeed, co-simulation environments play a key role due to the high risks of getting actual data from real scenarios in realistic driving and traffic conditions. 

## 3. Case Study with a Sensor Network in CPS for Driving Assistance: LiDAR On-Chip and GPS Sensors

A particular driving-assistance scenario is defined in order to evaluate and to validate the proposed co-simulation framework. The scenario emulates a real setup that is composed of a test track (a roundabout, traffic lights in the central crossing and additional curves on the main straight) that simulates an urban environment, a fleet of six fully-automated vehicles (distributed mobile nodes) and a main or central static node that is the communications tower [28].

In this case, a LiDAR sensor is modelled, specifically the 4-layer type Ibeo Lux. Table 1 shows the specifications of this sensor. The LiDAR specifications are inputs to the sensor model and distances between the detection points in relation to the vehicle position are outputs of the LiDAR model. Furthermore, the relative localization (X, Y, Z) of the detect points are outputs to the sensor model.

In addition, in order to determine the vehicle localization within the scenario, a DGPS 20 Hz receiver (Trimble BD960) is also modelled. In this model configuration, outputs of this model are the global coordinates of the vehicle location (latitude, longitude and altitude). Both sensor models are incorporated into the three vehicle models.

**Figure 5:**
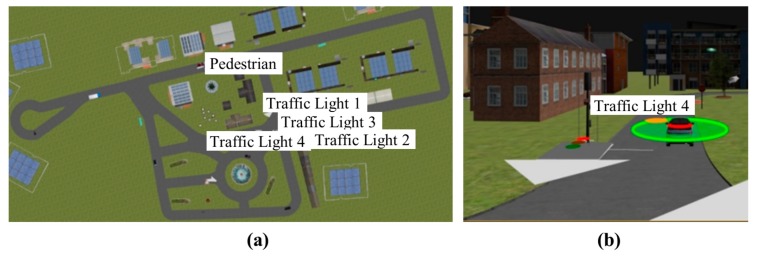
(**a**) Aerial view of simulation scenario of the CPS; (**b**) A fully automated vehicle model.

**Figure 6:**
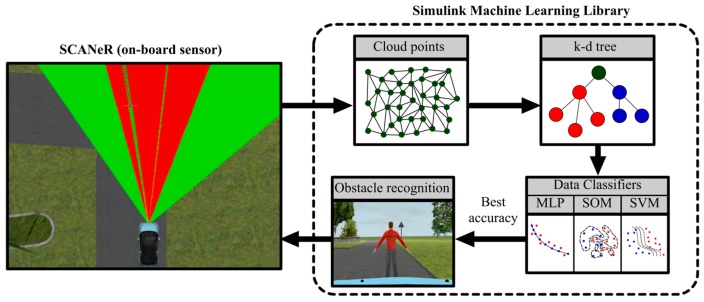
Obstacle detection close-loop for each virtual CPS sensor.

Once the CPS is defined, the next step is to define the object type to be identified in this particular simulation scenario. The virtual sensors mentioned earlier are incorporated in all vehicle models included in this scenario (Figure 5b), and can find different kinds of static and dynamic objects as obstacles—trees, traffic signs, traffic lights, vehicles, motorcycles and pedestrians—viewed from different directions and distances. For the sake of clarity, among common patterns in a real driving-assistance scenario, such as roads, lane markings, traffic signals, vehicles, pedestrians, and so on, only pedestrians were considered in this case study. The schematic diagram of the procedure to detect an obstacle for one virtual sensor in this particular use case is depicted in Figure 6.

### 3.1. Experimental Setup

The first step for implementing the obstacle-recognition library is to create a training data set from the information collected by virtual sensors using the Oktal SCANeR studio software v1.6. The scenario for simulation was set up with three fully automated vehicles (distributed mobile nodes) with the on-board particular sensors. In order to obtain the data set required to generate the classification models, data acquired from the LiDAR model (SCANeR module) were sent to the MatLab/Simulink module where these data were filtered, pre-processed and recorded. During this procedure, four hours of data provided by these sensor models were recorded. However, during the validation and evaluation of the obstacle-recognition library in the MatLab module, these models were applied to identify and classify obstacles. 

The analysis consists of a data-processing algorithm which begins with fitting the ground plane. It is necessary to search the ground plane and remove ground-plane points, using a RANSAC algorithm [29]. It is important to note that the weather considered in these simulations is a sunny day.

The next step during the data-processing algorithm is to extract the points that correspond to nearby obstacles corresponding to specific point-cloud sequence. Each scan of LiDAR data is stored as a 3D point cloud. In order to process the sensory data, fast indexing and search capabilities are required. The procedure is performed by means of “pointCloud objects” from the perception with the Computer Vision toolbox, which internally organizes data using a k-d tree structure [30]. After data processing, three subsets from the experimental population were yielded for training (70% of total samples), validation (15%) and testing (15%). For manually labelling the most relevant objects from the sensory information, the frames captured by another model sensor have been used. This model sensor is a stereo vision camera, the specifications of which are colour, 0.8 MP; resolution, 1032 × 776; and 20 FPS. The methodology is based on determining the region of interest in which the object to be classified is located in the image captured by the camera. On the other hand, the point cloud provided by the LiDAR is projected onto the image using a coordinate transformation, and only the points within the region of interest are selected.

The full dataset contains 1500 data subsets divided into 1050 segments or data subsets for training the classifier, 220 segments for validations and 230 segments for testing. The training part of the dataset contains 525 segments manually labelled positives (class-1 pedestrian detected) (LiDAR cloud points segments of pedestrian in upright entire body) and 525 segments without any pedestrian evidence (class-0, no detection of pedestrian). Instead, the validation part of data set contains 110 segments of positives and 110 negatives, also labelled (class-1 and class-0, respectively). Segments of the training and validation dataset contain 18 samples for the input with the corresponding output (class label) for each observation. Therefore, a matrix for each subset with *n* rows by 19 columns is generated, where *n* is the number of observations corresponding to each set. 

### 3.2. Training and Initial Test of Obstacle-Recognition Library 

As already mentioned, the library initially contains some classification models by default, and later, the content will be enriched in the runtime during the process simulation of the scenario. In this particular use case, three techniques are then considered, that is, a multi-layer perceptron neural network, a self-organization map and a support vector machine. The main rationales for their selection are their solid mathematical foundations, demonstrated ability in modelling in complex scenarios and a wide range of successful applications. The inputs of three classification models are datasets previously filtered and pre-processed (a point cloud) corresponding to the measurement provided by the LiDAR at each sampling time. Each dataset contains 18 input samples fed into the model. This maximum value of each dataset (i.e., 18 samples) is selected from simulations on the basis of the minimum number of samples that provides the necessary location information using the LiDAR. On the other hand, there is a single output of the classification model that corresponds to the object classification, that is, whether the object detected is a pedestrian or not.

The first technique was a multi-layer perceptron neural network with an input layer with 18 neurons, a hidden layer of 40 neurons and an output layer with a single neuron and linear activation function. For training, the method used was gradient descent with momentum and adaptive learning rate backpropagation. The initial values of learning rate and performance goal were 10^−7^ and 10^−8^, respectively. The network was trained during 50,000 iterations, after which it reached a best performance of 0.0216 and a gradient of 0.0021. Using the validation set, values of mean square error (MSE) of 0.0409 and correct classify samples or correct rate (CCR) equal to 95.91% were reached.

The second method for the obstacle-recognition library is a self-organizing maps method. Specifically, a topology function that creates neurons in an *N*-dimensional random pattern was used, and the dimensions were 22 × 2. Finally, the *Manhattan* function was applied as distance function. In addition, an input weight equal to the number of observations in the training set was set, that is, *w* = 1050. The network was trained during a cover step of 10,000 and an initial neighbour size of 4, after which it reached an MSE of 0.132, and a CCR equal to 89.55% was reached using the validation set.

Finally, a support vector machine was also implemented in the library. This nonlinear classifier uses a Gaussian kernel function with a kernel scale *σ* = 0.94 and a box constraint of 9.78 × 10^4^. The supervised learning method was trained during 1255 iterations, until its reason for convergence gradient reached a *Δ* < 0.001. The results obtained during validation were an MSE of 0.0636 and a CCR of 93.64%.

**Figure 7:**
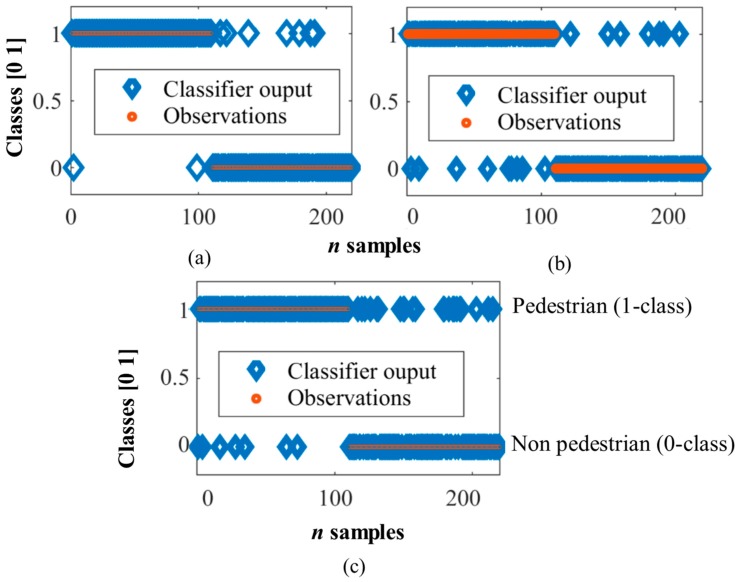
Validation results in pedestrian detection. (**a**) MLP; (**b**) SVM and (**c**) SOM.

Figure 7 shows the classifiers outputs of the three models vs observed classes using the validation set. The output of the classifiers is whether the detected object is a pedestrian (class 1) or not (class 0). The three classifiers showed very good performance indices, although the smallest error and the highest number of correctly classified instances corresponded to MLP, followed by SVM, and finally, the worse result corresponded to SOM. This study is not conclusive, and therefore, validation with an unknown data set is required (using more performance indices) in order to make a more complete comparative study among the three classifiers.

### 3.3. Final Validation of Obstacle-Recognition Library

The current testing, set in sunny weather conditions, contains 230 segments (i.e., 230 data subsets of 18 samples) and detailed annotations regarding the pedestrian appearances (in terms of occlusion), namely: occluded/partial pedestrians (class-0) and entire-body pedestrians (class-1). A summary of the testing dataset is shown in Table 2.

Six performance indices were considered in the validation study, on the basis of experimental run, as follows: correct classify samples or correct rate (CCR), incorrectly classified samples or error rate (ECR), the mean absolute error (MAE), the root mean squared error (RMSE), the relative absolute error (RAE) and the root relative squared error (RRSE). The results of the comparative study of the classifiers (MLP, SVM and SOM) are summarized in Table 3.

The application of the MLP yielded 23.64% of RAE. On the contrary, SVM and SOM achieved good accuracy on the basis of the RAE criterion, although not much lower in percentage than the MLP error. This good behaviour was also endorsed with high correctly classified sample rates of 91.36% and 90.91%. However, SOM and in particular SVM did not significantly outperform MLP with regard to all figures of merit considered in this study. It should be noted that this study was carried out in good weather conditions.

## 4. Experimental Results with Other Weather Conditions

Additional experimental tests, for evaluating the co-simulation framework and the performance of the library for obstacle detection in different weather conditions, were also conducted. Sunny, foggy, rainy and snowy conditions were taken into account in the conducted study.

The simulation time (2 h) for each weather condition is the same for virtual sensors in the CPS. All virtual objects and the corresponding positions are previously set. Some of these dynamic objects in the scenario are 205 pedestrians, 10 bicycles, 60 motorbikes, 213 small and medium vehicles, and 20 trucks. The goal is to assess the accuracy for detecting and identifying pedestrians in spite of the other obstacles that can be detected but not classified in this case study. Another test set was created for each weather condition (sunny, foggy, rainy and snowy). Each dataset consists of 1010 samples with 205 samples positively labelled pedestrian detections and 805 negatively labelled. The three classifiers were evaluated with these four datasets.

In order to assess the performance of the classifiers, the correct classify samples or correct rate (CCR) and incorrectly classified samples or error rate (ECR) were also calculated. Moreover, other performance indices were also considered, such as correctly classified positive samples/true positive samples or sensitivity (*Sn*), correctly classified negative samples/true negative samples or specificity (*Sp*), correctly classified positive samples/positive classified samples or positive predictive value (PPV), correctly classified negative samples/negative classified samples or negative predictive value (NPV), *Sn*/(1 − *Sp*) or positive likelihood (PL) and (1 − *Sn*)/*Sp* or negative likelihood (NL). The resulting performance indices (PI) for the four weather conditions (WC) are shown in Table 4.

In the case of CCR, there is a clear tendency to decrease the number of correctly classified instances due to the interference of weather conditions in the sensors’ fields of view. In sunny and foggy conditions, MLP and SVM showed better results than SOM. However, MLP showed a more evident deterioration with regard to the adverse weather conditions compared to SVM and especially SOM. The latter two remain more stable in spite of the weather conditions. In fact, SOM outperforms other topologies in the most extreme weather condition (snowy) with the highest specificity value (*Sp*). 

**Figure 8:**
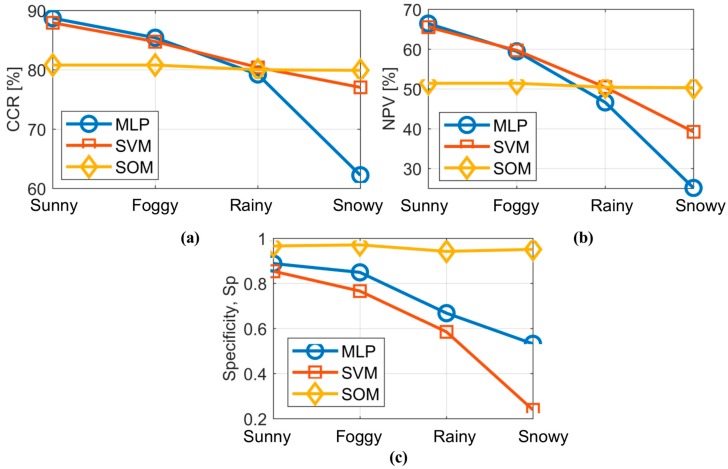
Behaviour of the performance indices for each classifier with regard to weather conditions. (**a**) CCR; (**b**) NPV and (**c**) *Sp.*

On the other hand, SVM produces the best classification in rainy conditions, although in sunny and foggy conditions, the results are worse than those given by MLP. Figure 8 depicts the behaviour of CCR, PPV and *Sp* of the three classifiers with regard to the four weather conditions.

It is evident that the best classifier differs according to the weather conditions. The classifier based on MLP behaves better than SVM and SOM for sunny and foggy conditions, whereas for rainy conditions, the SVM-based model is the most appropriate. However, for the most extreme weather condition (snowy), the SOM-based classifier is the most suitable. Overall, the SOM-based classifier depicts the most regular behaviour under all weather conditions.

## 5. Conclusions

This work presents a library of artificial intelligence-based methods for obstacle detection. The library is composed by three methods: a multi-layer perceptron neural network, a self-organizing map and a support vector machine. The library is integrated into a co-simulation framework for obstacle recognition on the basis of sensory data provided by a virtual sensor network in a cyber-physical system. This co-simulation framework is designed and built using SCANeR studio and Matlab/Simulink. Moreover, an assistance-driving scenario is created in SCANeR in order to represent and emulate the behaviour of a cyber-physical system.

The whole system is evaluated in a particular use case built from two types of sensory data (LiDAR on-chip and GPS sensors) within a defined scenario. The comparative study demonstrates that the proposed obstacle detection methods are powerful strategies for pedestrian detection. In the training and validation phase of the classification models, the best results were achieved with the multi-layer perceptron and the support vector machine, but the self-organizing map did not perform so badly as to be discarded from future analyses. 

In addition, a second evaluation is also performed, which consists of capturing sensory data provided by sensors in the presence of different weather conditions. In this second evaluation, all methods are able to adequately classify pedestrians. Multi-layer perceptron provides very good results in sunny and foggy conditions, but at the same time, has a tendency to deteriorate its performance in cloudy and snowy conditions. The support vector machine also produces the best result in rainy conditions. On the other hand, the self-organizing map produces the worst figures of merit, showing a more regular performance from data provided by all virtual on-chip LiDAR sensors. 

The results of this investigation corroborate the high influence of the weather conditions on the classifiers’ accuracy for detecting and classifying pedestrians. Nowadays, some initiatives in Europe, such as the ENABLE-S3, EMC2 and IoSENSE projects, are focused on highly automated cyber-physical systems in different domains with strong emphasis on automotive and transportation. Further research will be focused on an optimal tuning of the library methods, and the development of a self-organization procedure to select the most appropriate method among those available in the library in each particular scenario. 

## Figures and Tables

**Table 1:** Sensor model configuration. Virtual CPS sensor specifications (inputs to model).

Specifications/Inputs	Ibeo Lux 4 Layers
Horizontal field	120 deg. (35 to −50 deg.)
Horizontal step	0.125 deg.
Vertical field	3.2 deg.
Vertical step	0.8 deg.
Range	200 m
Update frequency	12.5 Hz

**Table 2:** Training and testing set for obstacle-recognition library implementation.

Full Data Set		Training Set		Validation Set		Test Set	
1500 segments		1050 segments		220 segments		230 segments	
Pos.	Neg.	Pos.	Neg.	Pos.	Neg.	Pos.	Neg.
750	750	525	525	110	110	115	115

**Table 3:** Comparative study of MLP, SVM and SOM.

Performance Index/Approach	MLP	SVM	SOM
CCR (%)	88.19	91.36	90.91
ECR (%)	11.81	8.64	9.09
MAE	0.12	0.09	0.09
RMSE	0.34	0.29	0.30
RAE (%)	23.64	17.29	18.68
RRSE (%)	9.274	7.93	8.36

**Table 4:** Comparative study of artificial intelligence-based methods under different weather conditions.

PI/WC	*Sunny*			*Foggy*			*Rainy*			*Snowy*		
	MLP	SVM	SOM	MLP	SVM	SOM	MLP	SVM	SOM	MLP	SVM	SOM
**CCR (%)**	88.70	87.92	80.79	85.40	84.75	80.79	79.21	80.40	80.00	62.22	77.03	79.90
**ECR (%)**	11.30	12.08	19.21	14.60	15.25	19.21	30.79	19.60	20.00	37.78	22.97	20.10
***Sn***	0.886	0.886	0.768	0.852	0.868	0.767	0.805	0.859	0.764	0.595	0.906	0.760
***Sp***	0.888	0.854	0.966	0.849	0.766	0.971	0.668	0.585	0.942	0.532	0.239	0.951
**PPV**	96.90	95.96	98.92	95.74	93.57	99.01	90.53	89.06	98.14	83.33	82.37	98.44
**NPV**	66.42	65.54	51.41	59.44	59.70	51.42	46.62	51.50	50.43	25.11	39.20	50.32
**PL**	7.894	6.05	22.48	5.635	3.709	26.19	2.427	2.073	13,05	1.271	1.19	15.56
**NL**	0.129	0.134	0.241	0.174	0.172	0.241	0.797	0.240	0.251	0.762	0.395	0.252
